# Nasolabial Cyst Associated with Odontogenic Infection

**DOI:** 10.1155/2016/8690593

**Published:** 2016-01-24

**Authors:** Eveline Claudia Martini, Fabiana Madalozzo Coppla, Eduardo Bauml Campagnoli, Marcelo Carlos Bortoluzzi

**Affiliations:** ^1^Department of Dentistry, State University of Ponta Grossa, Rua Carlos Cavalcanti 4748, Bloco M, Uvaranas, 84030-900 Ponta Grossa, PR, Brazil; ^2^Department of Stomatology, State University of Ponta Grossa, PR, Brazil

## Abstract

The nasolabial cyst or Klestadt cyst is a relatively uncommon nonodontogenic cyst that develops in the nasal alar region; it has uncertain pathogenesis. This lesion has slow growth and variable dimensions and is characterized clinically by a floating tumefaction in the nasolabial fold area around the bridge of the nose, causing an elevation of the upper lip and relative facial asymmetry. Diagnosis is primarily made clinically; if necessary, this is complemented by imaging. This paper reports the case of a 39-year-old male patient who complained of pain in the right upper premolar region and poor aesthetics due to a firm tumor in the right wing of the nose. Initially, this was thought to be due to an odontogenic abscess; however, the differential diagnosis was that a nasolabial cyst was communicating with the apex of teeth 14 and 15. Surgical treatment was carried out, followed by histopathological examination and concomitant endodontic treatment of the teeth involved.

## 1. Introduction

Nasolabial cysts can be considered as lesions that are not odontogenic and that affect the soft tissues of the nasal vestibule, canine fossa, and upper lip. These effects are unilateral to the midline of the upper lip and the base alar [1–4]. Nasolabial cysts are relatively rare, representing 0.7% of cysts affecting the maxillofacial region [5, 6].

The nasolabial cyst was studied by Klestadt in 1953 [2], and the lesion was named in his honor. Other names used for this lesion since 1941 include* mucoid cyst*,* maxillary cyst*,* wind cyst*,* nasovestibular cyst*,* subalar cyst*, and* nasoglobular cyst*. Neville et al. [3] and Choi et al. suggested the term* nasoalveolar cyst*, defining it as a lesion located between the soft tissues of the upper lip and nasal vestibule.

The pathogenesis of this lesion is uncertain, and there are several theories for its origin. The most accepted current theory is that the cyst originates from epithelial remnants trapped along the fusion of the lateral nasal processes with the median and the jaw; other theories speculate that it is a fissure cyst or that it originates from the deposition of the nasolacrimal duct epithelium [7].

Clinically, a nasolabial cyst is characterized by an increase in the volume of the unilateral nasolabial area causing an elevation of the bridge of the nose and superior lip projection [8]; in some cases, nasal obstruction occurs [2]. It presents as a slow and asymptomatic growth except when associated with an infection [9]. A nasolabial cyst typically forms between the first and seventh decades of life and is more common in adults aged 40–50 years, females (3 : 1) [1], and dark-skinned people [10].

The diagnosis is usually based on clinical outcomes and can involve imaging tests like X-ray, computed tomography (CT), and magnetic resonance.

The treatment of nasolabial cysts can be accomplished with a fine aspiration needle or through incision and drainage, cystic enucleation for intraoral access, marsupialization, use of endoscopic [8, 11] or sclerosing agents [4], or intralesional injection.

The aims of this paper are to present a case study of the treatment of a nasolabial cyst and discuss the diagnosis and characteristics of the lesion.

## 2. Case Report

**Figure 1:**
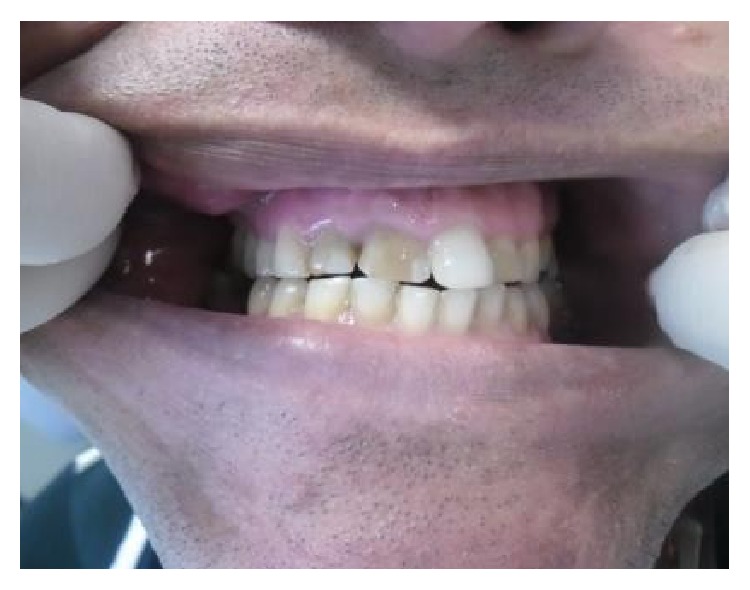
Initial dental appearance.

**Figure 2:**
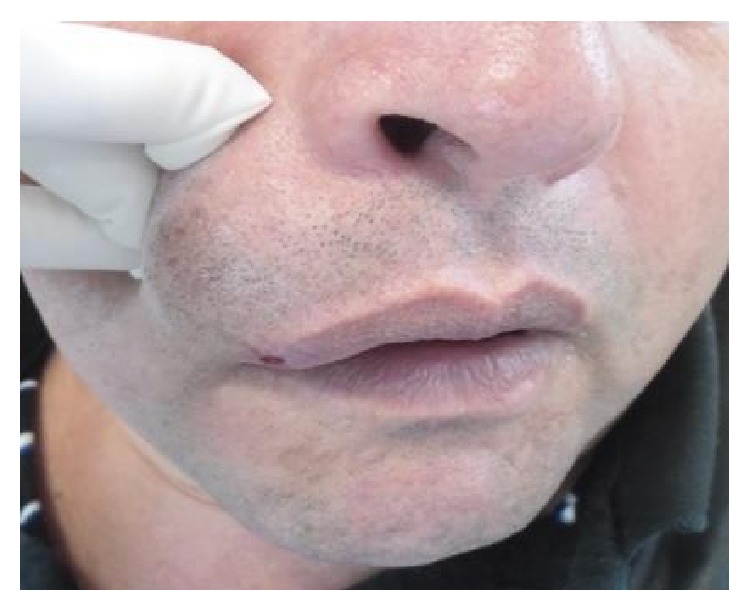
Initial view of the injury with external palpation of the face.

**Figure 3:**
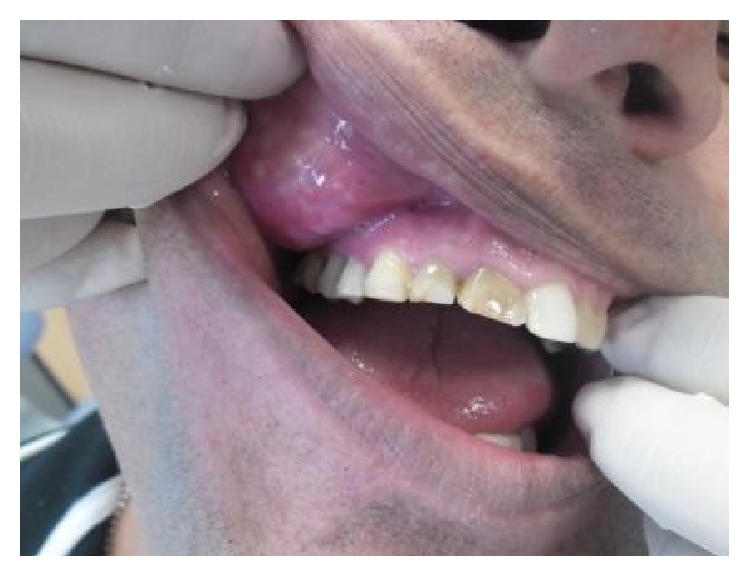
Initial view of the injury with internal palpation of the mucosa.

**Figure 4:**
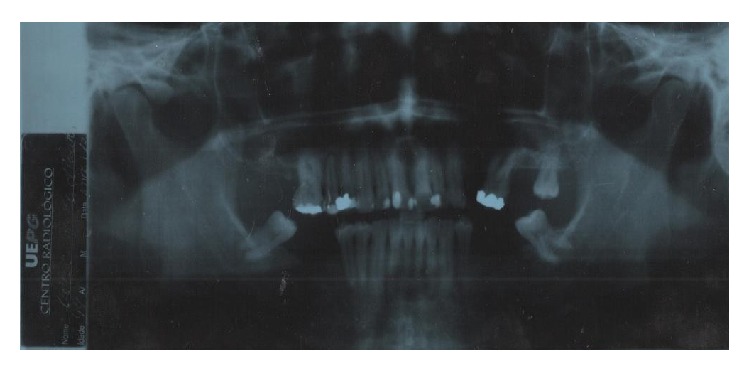
Panoramic radiograph of the patient.

**Figure 5:**
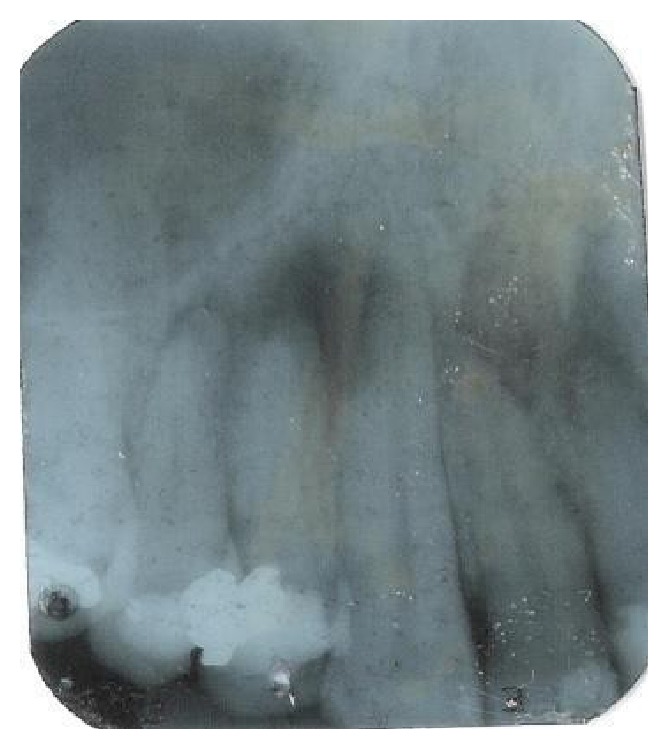
Periapical view of the right premolars. Extensive amalgam restorations on teeth 14 and 15 are observed; this location is close to the region of the apical periodontitis.

The patient, a Caucasian, 39-year-old male, entered the emergency department at the State University of Ponta Grossa complaining of an increase in the size of the paranasal sinuses on the right side and of diffuse pain in the teeth in the same region; the patient also complained of poor aesthetics due to swelling for several months. During the intraoral clinical examination, palpation revealed an increased volume close to the bottom groove region (Figures 1, 2, and 3). Panoramic (Figure 4), periapical (Figure 5), and occlusal radiographs of the maxilla were requested. Relevant to the radiographic images, there was apical periodontitis in teeth 14 and 15 that was compatible with an abscess.

Bringing together the clinical characteristics with the radiographic images, a diagnosis of endodontic abscess was made, for which the emergency procedure involved opening and endodontic instrumentation in teeth 14 and 15 and prescriptions for diclofenac potassium (50 mg for 2 days) and amoxicillin (500 mg for 7 days). The aim was to reduce local swelling and other symptoms. Two days later, the patient returned to carry out an exchange of intracanal medication and receive endodontic reinstrumentation on both teeth. After 8 days, as no decrease in the lesion was observed and the patient complained of pain, another exchange of intracanal medication was carried out. The following were prescribed: amoxicillin (875 mg) and clavulanate potassium (125 mg) for 7 days and dipyrone sodium (500 mg) for 2 days.

After another 7 days, the patient returned with similar symptoms. Fistula tracking with gutta-percha points was then made, followed by radiographic examination, which confirmed that the periapical lesions in question actually came from teeth 14 and 15. The intracanal medication was switched again, and a pulp sensitivity test was conducted on all upper teeth; beyond those in question, teeth 21, 11, and 12 also did not respond to the test. These results were forwarded to endodontics, since besides the sensitivity test these teeth were seen to be darkened, which is compatible with pulp necrosis.

Because endodontic treatment has not shown the desired effect or remission of early symptoms, complete blood count and fasting glucose have been invited to check systemic condition and immune ability of the patient. Nothing abnormal in laboratory tests was observed; a diagnosis of nasolabial cyst in the right facial region with possible communication with the roots of the teeth 14 and 15 was raised. After 1 week, a further change to the intracanal medication was made due to a lack of remission in the initial symptoms.

**Figure 6:**
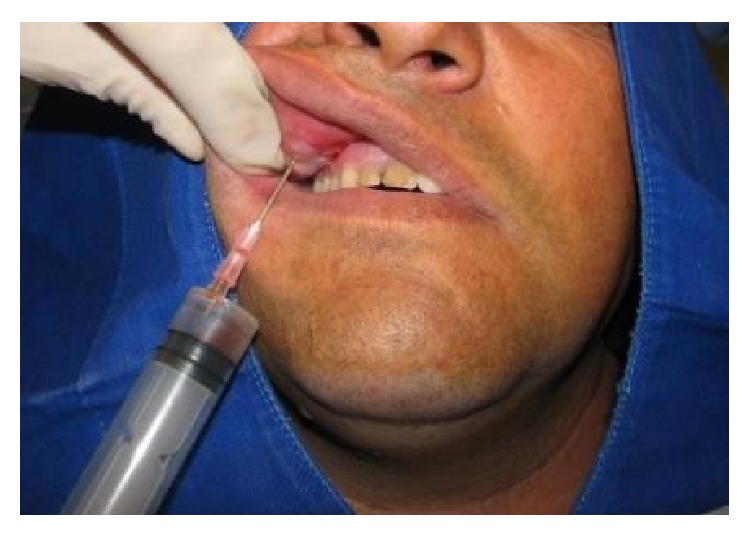
Aspiration of cystic content prior to surgery showed liquid with characteristics similar to that found in nasolabial cysts.

**Figure 7:**
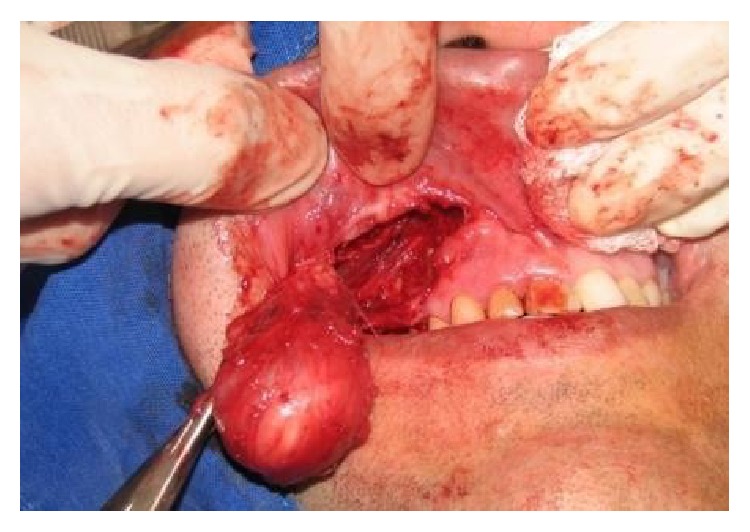
The view of the lesion; it was removed carefully because it was deeply embedded in tissues.

Given that after this period the symptoms had not deteriorated, surgical treatment of the case was then proposed. This was initially performed using aseptic face PVPI to prepare the surgical field after local anesthesia of the infraorbital nerve and the cellular anterior nasopalatine region using three tubes of mepivacaine (2%) and epinephrine (1 : 100,000). First, the cystic lesion contents were aspirated (Figure 6), followed by an excisional biopsy (Figure 7). The surgery had no complications.

**Figure 8:**
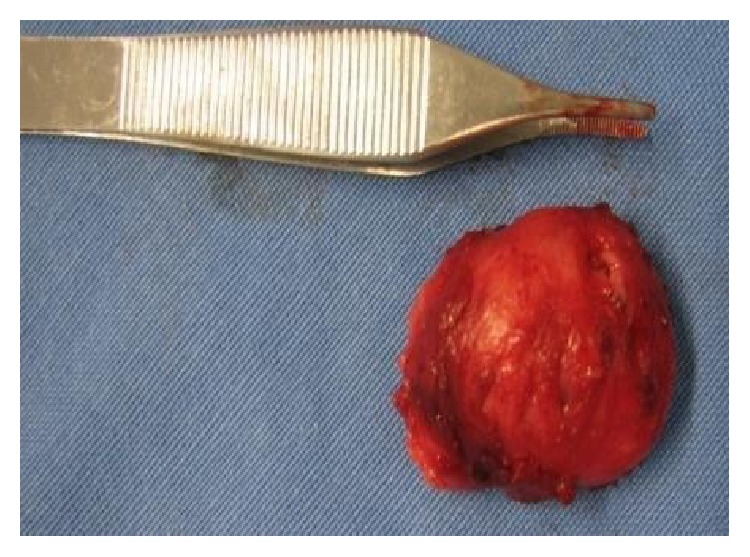
Appearance of the injury after complete removal.

**Figure 9:**
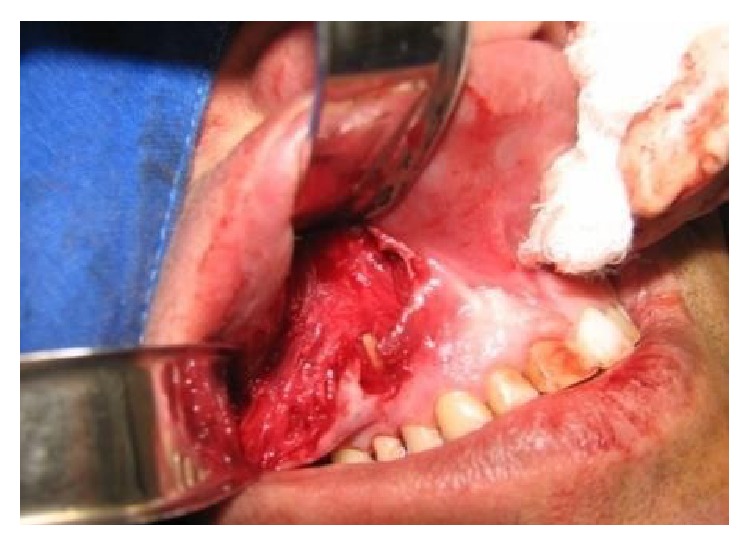
Note the close relationship of the lesion with the apex of premolars, which confirms the hypothesis of mutual periapical-cystic infection.

**Figure 10:**
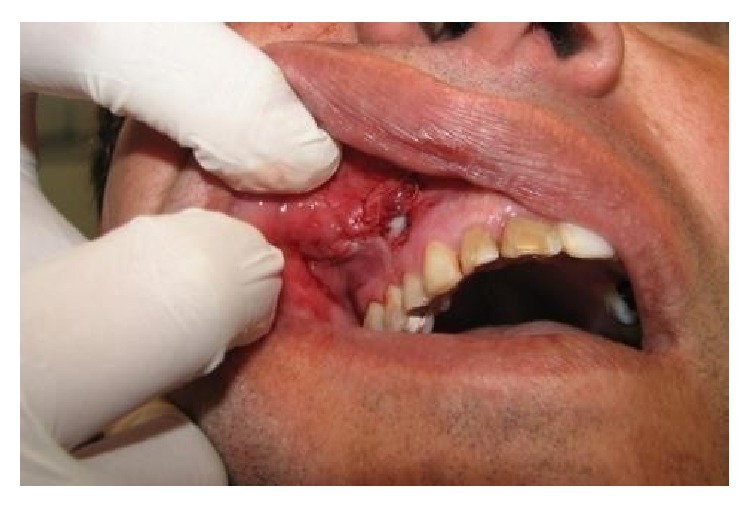
Appearance of the tissue after the end of the suture.

We found in the swelling an oval, whitish, noninfiltrating lesion (Figure 8); it had a smooth surface with defined borders, and it was hardened, sessile, and approximately 5 cm in diameter. After removal of the lesion, attention was drawn to the root of tooth 14, which had communication with the location of the cyst, confirming that there was a contamination relationship between the nasolabial cyst and the apical periodontitis, explaining why the endodontic treatment had not had the desired effect (Figure 10). The suture was performed (Figure 9) with Vicryl polyglycolic acid yarn (Janssen Pharmaceutica Ethicon Inc., New Brunswick, New Jersey, EUA).

**Figure 11:**
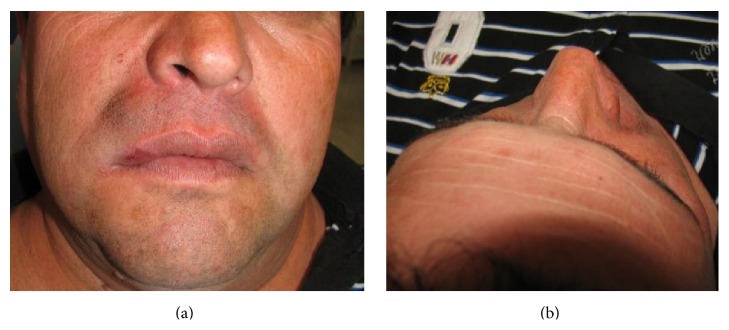
(a) and (b) Front and top facial views in the immediate postoperative period.

The lesion was properly stored and sent to clinical pathology for analysis, and the patient received a prescription for anti-inflammatory postoperative medication (600 mg ibuprofen for 3 days). The outer immediate postoperative appearance of the patient may be observed in Figure 11; this appearance was satisfactory, as the main complaint was the increase in the volume of the face region, and this was removed during the surgical procedure.

The clinical pathology analysis showed that the specimen consisted of a fibrous cyst wall with a light-coated cylindrical pseudostratified epithelium enclosing mucous-secreting goblet cells and inflammatory cells (due to the constant cystic contamination), which were seen communicating with the periapical region of the right premolars, as previously described. After examination, the diagnosis of a nasolabial cyst was confirmed.

**Figure 12:**
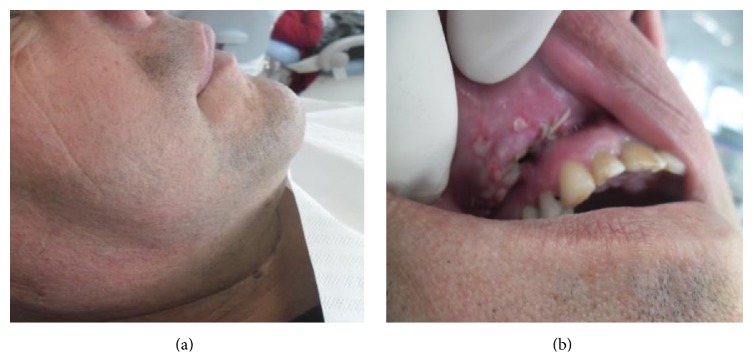
(a) and (b) Appearance of the face and the patient's mucosa in the postoperative period (after 7 days).

**Figure 13:**
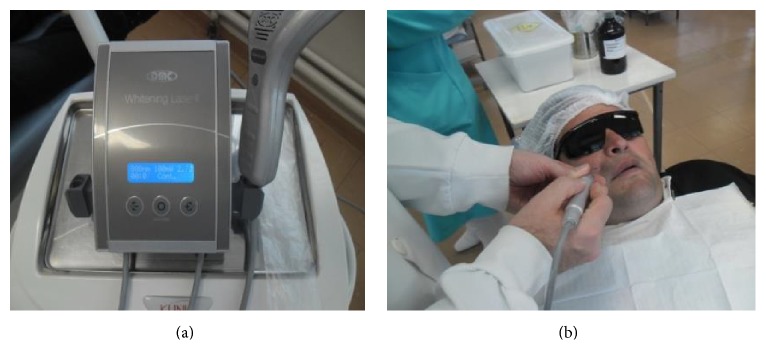
(a) and (b) Equipment of laser therapy and procedure performed in the region of the surgery 7 days after the chirurgic procedure.

**Figure 14:**
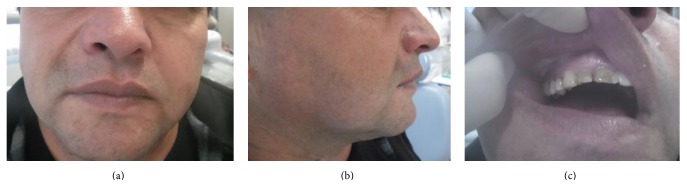
(a, b, and c) View of the patient's frontal and lateral face and mucosa 15 days after the operation.

Postoperative controls were made at 7 days (Figure 12), 15 days (Figure 14), and 1 month to evaluate possible volume increase and symptom recurrence in the region, including site pain, paresthesia, or any other signs of neurological changes in the region. At the first postoperative visit, paresthesia of the nasolabial and labial region was observed; these sites were listed for laser therapy sessions (Figure 13) aimed at recovering sensitivity in the region. The patient has conducted 2 sessions of laser therapy; after the second one, the patient reported that he was beginning to feel the surgical site.

## 3. Discussion

This case report illustrates the most common features found in patients affected by nasolabial cysts, which corroborates previous studies [4, 6, 7, 12]. Some common clinical signs observed here, such as the deletion of the nasolabial folds and the elevation of the nasal wings, could have led to the final diagnosis upon first contact with the patient; however, the story reported by the patient made it difficult to diagnose an injury that did not involve bone tissue. This difficulty can lead to a demand for various professionals to ensure the complete resolution of the case [10].

The patient reported growth of the injury within a few months, along with painful symptoms, unlike what is commonly found [2, 6, 8], and he was complaining about poor aesthetics due to bulging in the region. This differed from the literature [6], in which some patients reported development of this lesion over a period of 3 to 5 years and did not seek treatment due to its slow, asymptomatic growth and the absence of pain and discomfort. In other cases [13], there was a sudden growth of the lesion after 1 year, and in one case, after a period of only 2 months, the lesion became excessive.

Regarding the preference for race, the present case also differs from the literature, as dark-skinned people have shown the highest number of occurrences with respect to age and gender [12, 14]. In this case, the lesion was diagnosed unilaterally after a thorough evaluation of the opposite side and a pulpal vitality test of the teeth in quadrant 2. There are few cases of bilateral nasolabial cysts in the literature.

The diagnosis of nasolabial cysts is almost exclusively clinical, in which bidigital palpation of the region is essential and should be careful [2, 6, 12]. What hinders the process is that it is a relatively rare lesion and that the differential diagnosis can include many other conditions affecting the anterior maxillary region, including odontogenic cysts, periapical granulomas, and abscesses. The pulp vitality test of the adjacent teeth is essential for a proper diagnosis, and it must first be carried out, as if it is not apical periodontitis, the teeth in question will show positive results for vitality in the presence of nasolabial cyst. This feature made it difficult to diagnose this case, as the clinical characteristics of the patient matched with apical periodontitis and the teeth in question responded negatively to the pulp vitality test.

Dermal and epidermal cysts should also be considered in the differential diagnosis, although these are associated with the yellowing of the overlying mucosa; in cases of nasolabial cysts, they hold to your labial mucosa with normal color or bluish color, due to the presence of local vascularization and depending on their size [7, 12, 15]. In addition, epidermoid cysts are usually diagnosed in childhood, while nasolabial cysts are more common in adult patients [12], as was noted in this case.

El-Din and el-Hamd [6] reported that complications of the injury, when occurring, generally cause nasal obstruction and appearance of cosmetic redness on the patient's face. According to Nixdorf et al. [13], patients only seek therapy when there is deformity, nasal obstruction, or an infection caused by the lesion [5, 9, 15]. However, this patient sought treatment because he thought the injury was of dental origin, as there was a communication of the nasolabial cyst with the root tips of teeth 14 and 15.

Regarding treatment of the injury, most studies have reported enucleation as the treatment of choice for nasolabialis [2, 4, 5, 8, 10, 12] and cysts, as they are composed entirely of soft tissue, which does not respond well to marsupialization. Alternative methods of treatment have been suggested, including aspiration, cauterization, injection of sclerosing agents, and incision with drainage. However, these methods are associated with higher rates of relapse [3, 11, 12, 15]. Therefore, in view of the ease of surgical resection and its curative potential, we believe this procedure should be the treatment of choice in most cases. For this patient, despite the size of the lesion, we chose to perform surgical removal after a histological study of the region.

As for the cyst aspiration procedure, which aims to assess the possible presence of cholesterol crystals, remove odontogenic injuries, and enable injection of radiopaque solution, this method was contraindicated by some authors because it may increase the risk of lesion recurrence [12–14, 16], especially when it is chosen as the only form of treatment. In this case, this was only an auxiliary part of the diagnosis; therefore it could be used.

**Figure 15:**
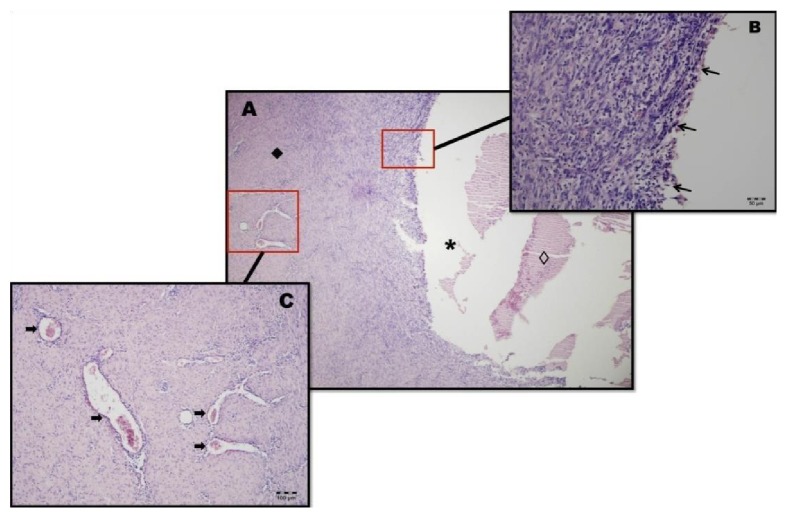
(A) View of the cystic nature of the lesion; the cystic cavity (*∗*) had the presence of necrotic material (◊), and the cystic capsule was found to be quite thick (◆) (HE, 40x). (B) View of the destruction of the epithelial lining demarcating the cystic cavity (arrows) and the presence of intense inflammation associated with granulation tissue (HE 200x) due to recurrent episodes of infection associated with cystic tooth infection. (C) View of the cystic capsule, which has been collagenized and cellularized and shows several blood vessels that are dilated and have an accumulation of erythrocytes inside (arrows) but no atypical cells (HE, 100x).

In our patient, the histopathology was not entirely consistent with what we usually see in the histology of nasolabial cysts (Figure 15); due to a process of chronic recurrent inflammation, the cyst eventually lost some of its epithelial characteristics. However, we did observe characteristics of a cystic lesion with chronic inflammation signals, a fibrous capsule, bright and smooth inner surface, and dyed yellow seromucous content; therefore, through the combination of histological findings and clinical features found in the lesion the diagnosis of a nasolabial cyst was confirmed.

## 4. Conclusion

This paper presented clinical characteristics and histological findings consistent with a nasolabial cyst despite the framework that was initially described as being only of dental origin. This soft tissue injury should always be considered in the differential diagnosis when there is swelling in the soft tissue of the nasal alar region. The treatment in this case covers endodontic conduct in conjunction with surgical excision of the lesion; the treatment was conservative, as this type of lesion is a rare recurrence.
